# Naive, captive long-tailed macaques (*Macaca fascicularis fascicularis*) fail to individually and socially learn pound-hammering, a tool-use behaviour

**DOI:** 10.1098/rsos.171826

**Published:** 2018-05-09

**Authors:** Elisa Bandini, Claudio Tennie

**Affiliations:** 1 School of Psychology, University of Birmingham, Birmingham B15 2TT, UK; 2 Department for Early Prehistory and Quaternary Ecology, University of Tübingen, Tübingen 72070, Germany

**Keywords:** zone of latent solutions, individual learning, social learning, pound-hammering, *Macaca fascicularis aurea*, *Macaca fascicularis fascicularis*

## Abstract

A subspecies of long-tailed macaques (*Macaca fascicularis aurea; Mfa*) has been reported to use stone tools and a specific technique to process nuts in Southeast Asia, a behaviour known as ‘pound-hammering’. The aim of this study was to examine the development of pound-hammering in long-tailed macaques: whether this behavioural form can be individually learnt or whether it has to rely on some forms of social learning. Given the absence of *Mfa* from captivity, long-tailed macaques of a highly related subspecies (*Macaca fascicularis fascicularis; Mff*) were experimentally tested by providing them with the ecological materials necessary to show pound-hammering. A baseline was first carried out to observe whether pound-hammering would emerge spontaneously without social information. As this was not the case, different degrees of social information, culminating in a full demonstration of the behaviour, were provided. None of the subjects (*n* = 31) showed pound-hammering in any of the individual or social learning conditions. Although these data do not support the hypothesis that individual learning underlies this behaviour, no evidence was found that (at least) *Mff* learn pound-hammering socially either. We propose that other—potentially interacting—factors may determine whether this behaviour emerges in the various subspecies of long-tailed macaques, and provide a novel methodology to test the role of social and individual learning in the development of animal tool-use.

## 1. Introduction

Long-tailed macaques (*Macaca fascicularis*) are commonly found throughout Southeast Asia and have been classified into 10 different subspecies following genetic, anatomic and geographical differences [1]. Studies have mostly focused on *Macaca fascicularis fascicularis (Mff)*, due to their widespread distribution in Southeast Asia. More recently, *Macaca fascicularis aurea (Mfa)* has received increased attention due to the scientific (re)discovery of complex stone-tool-use practices within some *Mfa* populations [2–4]. *Macaca fascicularis* have a flexible diet that allows them to exploit several different encased food sources such as nuts and shelled marine prey [4,5]. Four out of the eight currently observed populations of Burmese long-tailed macaques (*Mfa*), including one population of hybrid *Mfa* × *Mff* individuals [1], flexibly process shelled foods, such as rock oysters (*Saccostrea cucullata*), crustaceans, molluscs (e.g. gastropods and bivalves) and nuts, including sea almonds (*Terminalia catappa*) and oil palm nuts (*Elaeis guineensis*) [5] using various stone tools and techniques [1,4]. To open sessile rock oysters, *Mfa* individuals have been observed to adopt a more controlled ‘axe hammering’ technique, in which a small hammer stone is used to crack open attached valves [6]. Detached food sources, such as gastropods and sea almonds, are processed using another strategy, ‘pound-hammering’, in which the items are brought to an anvil (generally a large standing stone) and cracked open with a stone hammer [4]. These tool-use behaviours have been extensively recorded for some *Mfa* communities and despite the close spatial and genetic relationship between subspecies, no instances of using stones to crack open objects have been observed in the other subspecies of long-tailed macaques [1].

Although pound-hammering has been rigorously recorded for *Mfa* communities in the wild, very little is known about how the behaviours first emerge throughout *Mfa* and the hybrid populations, and why this behaviour is not practised by *Mff.* Understanding how these tool-use behaviours develop across individuals may provide explanations as to why they are confined to only some populations and subspecies.

Several primate species use tools [7]. However, to date, primate *stone* tool use has only been recorded in chimpanzees [8], capuchins [9–12] and long-tailed macaques [2–4]. Until recently, most reports on wild (non-human) primate stone tool use have been of stone hammers being used to crack open shelled food sources. Yet, the recent observation of wild bearded capuchins (*Sapajus libidinosus*) deliberately breaking stones—possibly in order to ingest powdered quartz and/or lichens [12]—is an exception. Due to the similarities between stone tool use techniques of extant non-human and human primates [13], data from these studies may allow for inferences to be made on the techniques, behaviours and cognitive mechanism involved in the evolution of stone tool use in the hominin record [5].

The aim of the current study was to observe whether the overall behavioural *form* of one of these behaviours, pound-hammering, is the product of spontaneous individual learning and only its frequency realized by various low-fidelity social learning mechanisms, or whether the behavioural form itself is and has to be socially learnt and transmitted (currently the predominant theory for primate tool-use behaviours; e.g. [14–21]). The current study focused only on the pound-hammering technique, which is arguably more complex than axe hammering as it requires three interactive objects: stone tool, food source and substrate stone as an anvil [22]. Conversely, the alternative technique, axe hammering, involves only two objects: a stone and the attached food source.

To identify the mechanisms involved in the emergence of pound-hammering, the latent solutions testing methodology (LS test, part of the zone of latent solutions (ZLS) hypothesis [23,24]) was applied to naive *Mff* individuals, who had never seen the pound-hammering behaviour before. We also developed and extended this testing methodology here. In the original case, LS tests involve providing naive individuals with all the ecological materials for the behaviour, and no demonstrations of the target behavioural form, to examine whether the form will arise spontaneously—logically this would then have to be through individual learning. The first condition, in which no social demonstrations of the behavioural form are provided, will be here referred to as the ‘baseline’ condition. If subjects individually express the same behavioural form in the baseline condition, this demonstrates that the behavioural form is within the species' individual learning abilities. If so, the logical conclusion is that social learning is not *required* for the behavioural form to emerge (a standard biological principle). However, various forms of social learning (see [25] for an overview of the different types of social learning) may facilitate the *frequency* of the behavioural form in a population (through low-fidelity forms of social learning, i.e. forms unable to transmit the whole behaviour itself, but able to increase the likelihood of the behaviour emerging in affected individuals^1^). Thus, the LS test allows for the role of individual learning and the *necessity* of social learning to be identified in the emergence of behaviours. As pound-hammering did not develop spontaneously in the naive *Mff* tested in this study, various levels of social learning were provided to examine whether social information would facilitate the acquisition of the behaviour. Although this was not the case, and the *Mff* never developed pound-hammering, we successfully applied our new testing methodology to identify the roles of various forms of individual and social learning mechanisms in the emergence of a novel tool-use behaviour. This testing methodology can be applied to all animal behaviours to identify the learning mechanisms involved, and can be used to experimentally test the ZLS hypothesis for particular behaviours [23]. We present the results of this study with *Mff* and the novel testing methodology here.

## 2. Methods

### 2.1. Latent solutions testing methodology

The baseline condition, in which the only stimuli provided are the materials necessary for the behaviour to be performed, may not be sufficient to encourage the acquisition of the target behaviour. One limiting factor is that subjects may not be motivated enough to express the behaviour (because, for example, they receive regular daily feeds). Thus, the first part of our updated LS testing methodology proposes a stepwise approach, in which the amount and type of information provided to subjects is gradually increased—though never includes demonstrations of the behavioural form. This allows for control over the amount and type of social learning provided in order to identify what conditions are required for the subjects to individually derive the target behavioural form.^2^

### 2.2. Results-dependency testing conditions

As action information is necessarily hierarchically placed above result information [26,27], and most importantly, actions prescribe the behavioural form (i.e. our target) itself, target action information should, ideally, only be demonstrated at the very end of the sequence of conditions. Given several similar previous tests in the literature (carried out for different reasons, e.g. [28,29]), the first set of conditions we used after the first LS baseline provide information on the end results of the behaviour (i.e. the physical static final environmental result of the behaviour). The first low-fidelity social learning condition we used involved a ‘*partial* end-state condition’, in which only part of the environmental outcomes that typically would result from the target actions were demonstrated (but without actually demonstrating these actions). The following condition, the ‘results-dependency condition’, tested for the reproduction of complete end-results (yet still without revealing the actions that were used to achieve this end-state). The final condition in the result-dependency test set involves an ‘object movement re-enactment condition’, in which the relative movement of the objects involved (stone to nut) *as well as* the overall end-result are shown (yet, again, this condition does not involve an agent demonstrating action information; a so-called ‘ghost condition’ [30–32]). The object movement re-enactment condition therefore additionally reveals the sub-results and their relationship to each other, but still not the target actions. Note that, until this point, no target action demonstrations are provided. If the target behavioural form emerges in any of these conditions, these actions would have to be derived through individual learning and their expression may only have been aided by low-fidelity social learning, as the crucial actions required for the behavioural form to emerge are never revealed.

### 2.3. Action-dependency testing conditions

If the target behaviour does not emerge in any of the LS test conditions, the next step is to provide demonstrations of the actions required for the behaviour (i.e. to demonstrate the full form of the behaviour: especially the actions, but also the results of the behaviour). Thus, the following conditions include full action demonstrations of the target actions, results (including the end state) and even the target goals of the behaviour in question (social learning conditions that allow for action copying, potentially allowing for the copying of the full behavioural form itself) in order to assess whether the behaviour is a culture-dependent trait (i.e. action traits that cannot be individually learnt [29]). Only in cases where a behavioural form first does not emerge in the LS test conditions, but then later emerges in the action-dependency testing conditions, can this behavioural form parsimoniously be considered to require social learning.

The new testing methodology presented here therefore allows for the learning mechanisms involved in the emergence of a new behavioural form to be isolated and identified.

### 2.4. Pound-hammering test

To the best of our knowledge, the subspecies *Mfa* (i.e. currently the only subspecies that demonstrates pound-hammering in the wild [1]) is not found in captivity. Thus, all tests were carried out with *Mff.* We decided to proceed with testing this subspecies as *Mff* are very closely genetically related to *Mfa*, and in particular because the hybrids of *Mff* and *Mfa* have already been observed to use tools in similar ways to *Mfa* [1]. By testing *Mff* our data therefore would generate information on two levels: positive evidence for individual learning of actions (in the LS test) underlying pound-hammering in *Mff* would simultaneously show that the behavioural form can emerge in the absence of social learning for *Mff* and that high-fidelity social learning is unlikely to be necessary for wild *Mfa* to express the behavioural form as well. The former is a possibility despite wild *Mff* not (yet) having been reported to show this behaviour (also because captive animals may be more likely to show tool-use behaviours than their wild counterparts, due to the phenomenon known as the ‘captivity effect’ [33,34]).

The updated LS testing methodology—alongside the action-dependency tests—was applied to two populations of *Mff* naive to pound-hammering held at two wildlife parks in the UK (*n* = 31, *M*_age_ = 19.3). Following the results of previous tests on the individual learning abilities of primates (e.g. great apes [35–42]; capuchin monkeys [9,43]; and even in long-tailed macaques [44]) pound-hammering was hypothesized to emerge in naive individuals within the first baseline condition. As this was not the case, we proceeded along the series of test conditions according to the updated testing methodology, as detailed above.

### 2.5. Statistical power for latent solutions tests

As findings from captive populations need to be generalized to make predictions on a species-wide level, it is essential that LS tests have the required statistical power to generalize data from samples to the whole species. Here we update the LS test methodology with regard to this point. Note that the below is only applicable for test conditions in which the target behaviour does *not* occur.

According to Cohen [45], statistical power of an experiment should aim to exceed 80% to allow for confident conclusions to be drawn from datasets. By ‘power’ here we refer to the probability of observing the target behaviour in at least one, or two, individuals. Following this guideline, we propose that LS tests *failing* to detect a target behaviour must exceed 80% power in order to confidently draw conclusions from a specific sample size (which we calculated below) to a species-wide level. In addition, we propose two such standards for LS tests. Relatively complex behaviours (e.g. nut cracking in chimpanzees, which requires the use of at least two tools (an anvil and a hammer, both of which may be of different materials [8]) and a sequence of actions and placements which must be followed in order to successfully crack the nuts) require only *one* observation to conclude that the behaviour can emerge through individual learning. These multi-tool and multi-step behaviours are *relatively* complex and therefore unlikely to occur simply by chance alone, even once (single-case standard [41]). Thus, we propose that for relatively complex behaviours such as this, the expression of the behaviour by a single naive individual suffices to classify the behaviour as within the individual learning abilities of the species (indeed, spontaneous nut-cracking has already been identified in captive chimpanzee groups [46,47] and in a group of captive capuchins [9]). On the other hand, remaining with the example of chimpanzee tool use, some behaviours are relatively less complex—for example, stick tool use in chimpanzees (e.g. using a stick to retrieve bone marrow from long bones [15] or honey [48]; although see also [49] for an alternative view on the complexity of stick tool use in chimpanzees). Inserting a stick into an appendage or hole to retrieve food involves fewer placements and object relations than the relatively more complex nut-cracking. Thus, due to the relative simplicity of the actions and relationships among objects required for less complex behaviours such as stick use, there is a more realistic chance (albeit still a small one) that the behaviour may appear once through non-purposeful acts (e.g. during displays, or by mistake). Therefore, to ensure that the behaviour observed truly emerged via individual learning, these simpler behaviours must pass the double-case standard, in which *two* independent individuals must show the behaviour before it can be confidently assumed to be in the individual learning abilities of the species [40,41].

Once the behaviour has been classified as relatively simple or complex, the required minimal sample size needed to confidently draw conclusions from the data can then be calculated (following Cohen's [45] requirement of at least 80% power, see above). We carry out this calculation below, based on binomial cumulative distributions. To calculate the minimum sample size, the expected probability of individual innovation is also required. If the behaviour can be individually learnt, and the subject is motivated to engage with the situation, we hypothesized that the probability of acquisition in a given individual of the target behaviour (i.e. outside high fidelity social learning) must fall within a range that reaches from very high (100%—in case of behaviours that may not even need to be harmonized by low fidelity social learning in a population^3^) to low (but not very low: the target behaviour should occur in independent naive individuals with a probability that must substantially exceed zero; C. van Schaik 2016, personal communication). For current purposes, to derive at minimally required sample sizes, we only need to define the lowest estimates of this probability. Given these considerations, and the fact that the empirically derived rates of pure individual innovation so far seen in latent solution experiments were relatively high ([37] (83% expression rate); [38] (at least 15%); [39] (at least 13%); [40] (80%); [41] (at least 14%)), here we propose a conservative standard of a (at least) 10% probability of pure individual expression of the behaviour (this being a low, but not very low rate already takes into account that we can expect raised motivation, and hence increased acquisition rates in captivity due to the captivity effect [34]). Thus, within both the single-case and the double-case standards, a 10% probability of acquisition of the behaviour is applied below. Given that both standards (see above) differ in the number of minimum observations (1 versus 2), they require a different sample size each to reach a power of 80%. Calculating this sample size shows that, to reach a power of 80%, the single standard requires a sample size of at least 16 subjects, and a sample size of at least 29 subjects in the double standard. The minimum sample size is calculated using a binomial cumulative distribution (once the required expression rate and the probability of acquiring the behaviour are established; see electronic supplementary material for extended calculations):

$$F(k;n;p)=\mathrm{Pr}(X\leq k)=\overset{n}{\underset{i=0}{\sum }}\left(\begin{array}{c} n\\ i \end{array}\right)p^{i}{\left(1-p\right)}^{n-1}.$$

As this study examined the acquisition of pound-hammering, a relatively complex multi-step tool-use behaviour [2–4,50], the single-case standard was applied. Our sample size (*n* = 31) exceeded both the single-case and the double-case standard requirements.

## 3. Materials

### 3.1. Subjects

Two adult female long-tailed macaques (*Macaca fascicularis fascicularis*) held at Shepreth Wildlife Park, Cambridge, UK (*n* = 2, *M*_age_ = 22) and 29 long-tailed macaques (*Macaca fascicularis fascicularis*) held at Curraghs Wildlife Park in the Isle of Man, UK, participated in this study (*n* = 29, *M*_age_ = 16.7; 17 females; all captive-born). The first test was carried out at Shepreth Wildlife Park. The subjects were mother, Tina and daughter, Tammy. Tina was originally purchased by a private individual, and was donated to Shepreth Wildlife Park in January 1991. Tammy was born in captivity and reared by her mother at Shepreth Wildlife Park. Testing was carried out in November 2015 by E.B. As both individuals lived almost exclusively (except for Tina's first year) at Shepreth Wildlife Park, we could control for their past experience with similar tasks (see below). The second test was carried out at Curraghs Wildlife Park. This group of long-tailed macaques (*n* *=* 29) consisted of individuals ranging from infants (born at Curraghs Wildlife Park in September/November 2015) to older adults (*M*_age_ = 16.7) of both sexes.

### 3.2. Previous tool-use knowledge

All the keepers at both institutions filled out a questionnaire on the previous experiences of the subjects of any tasks that resembled the one presented in this study. The questionnaire was followed-up with interviews by E.B. with the keepers, in order to fully understand the previous knowledge of the individuals. The keepers from both parks reported that although the subjects received nuts occasionally, at Shepreth Wildlife Park, these were always unshelled (and therefore do not require any processing) and at Curraghs Wildlife park the animals were occasionally provided with shelled nut types, which they can easily crack with their teeth, or for the larger nuts (coconuts), the macaques cracked them by dropping them from the trees and hanging support structures in their indoor and outdoor enclosures onto the ground. No other shelled foods were ever included in any of the subjects' diets. Furthermore, the keepers reported that they never demonstrated the cracking action required to open nuts at either park, and that the animals were never involved in experiments or enrichment exercises that required tools to crack open objects. Although stones are found in the outdoor enclosures of both parks, the keepers confirmed that they have never observed the animals using the stones to crack open any objects. Thus, we could conclude that the individuals were naive to pound-hammering before testing.

### 3.3. Procedure

#### 3.3.1. Shepreth Wildlife Park testing

**Figure 1.**
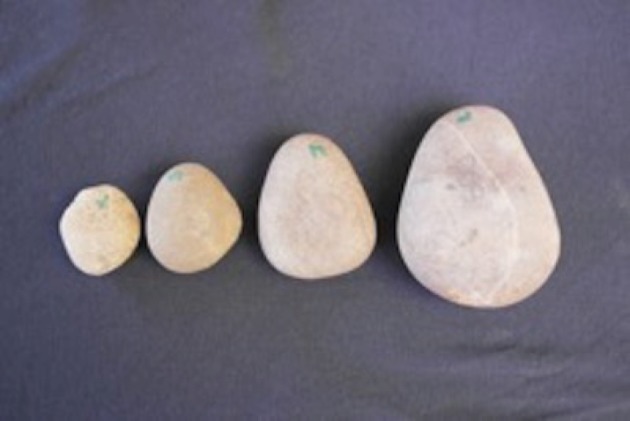
The four stones ranging from X to L placed inside the subjects' enclosure (photograph by E.B.).

The subjects at Shepreth Wildlife Park were provided with the relevant stone tools and food sources to enable pound-hammering (figure 1). Tools consisted of four stones ranging in mass following previous findings [51–53] on tool mass selection according to food type: X (40–60 g), S (90–100 g), M (150–200 g) and L (400–1000 g). Despite large standing stones being available in the enclosure, an anvil stone (with one large, flat surface and nooks) was also provided (2000 g). The stones and anvil were placed near the fence in the outdoor enclosure by the keepers before allowing the subjects back in. On the first 2 days of testing, raw, live clams (Mollusca: Bivalvia) were placed inside the enclosure by the keepers. Neither subject showed interest in the clams, therefore the clams were replaced by hard nuts: encased unroasted macadamia nuts (*Macadamia integrifolia*) thereafter (all the subjects showed an interest in these nuts).

The items were placed in the outdoor enclosure by the keepers before the subject was allowed into the area. The two subjects were kept separately while testing to control for social learning effects in case one individual expressed the behaviour spontaneously. Each testing session lasted 30 min and was filmed with a handheld Sony HDR-CX330E handycam. Over 35 h of observational data were collected from Shepreth Wildlife Park.

#### 3.3.2. Curraghs Wildlife Park

The same procedure as at Shepreth Wildlife Park, other than the differences described below, was carried out at Curraghs Wildlife Park with 29 subjects in March–May 2016. As the first two individuals at Shepreth Wildlife Park did not show an interest in the clams, the Curraghs Wildlife Park long-tailed macaques were immediately provided with macadamia nuts. Furthermore, the same sea almonds (sourced from Thailand) that wild *Mfa* process in Thailand [6,54] were included alongside the macadamia nuts. Fifteen macadamia nuts and seven sea almonds were provided in each testing session. The individuals at Curraghs Wildlife Park could not be individually separated so were tested as a group. All subjects from Shepreth Wildlife Park and Curraghs Wildlife Park had free access to their indoor and outdoor enclosures. Although all the 29 individuals from Curraghs Wildlife Park were in one group, there were two semi-independent social groups within this group. Around 80 h of video data were recorded at Curraghs Wildlife Park. All subjects participated in five testing sessions (30 min each) per condition (see below) over a period of six weeks.

### 3.4. Conditions

We tested subjects across different conditions (see Introduction).

#### 3.4.1. Baseline condition

This first condition tested for unprompted, spontaneous individual acquisition of the behaviour—without the help of any type of social learning. The stones were placed inside the enclosure before allowing the subjects in. No demonstrations were provided. To ensure that the subjects would not reject the nuts, a keeper at Shepreth Wildlife Park consumed store-bought unshelled macadamia nuts in front of the subject while handing them unshelled macadamia nuts. This process was repeated five times in total per individual. Both subjects ate the five nuts provided, thus confirming that this was a desirable food source. As the subjects at Curraghs Wildlife Park had received and eaten unshelled nuts (including macadamia nuts) in the past, they were provided with the shelled macadamia and sea almonds without keeper-facilitation. All groups of long-tailed macaques received five 30 min sessions in total.

#### 3.4.2. Results-dependency testing conditions

##### 3.4.2.1. Partial end-state condition

**Figure 2.**
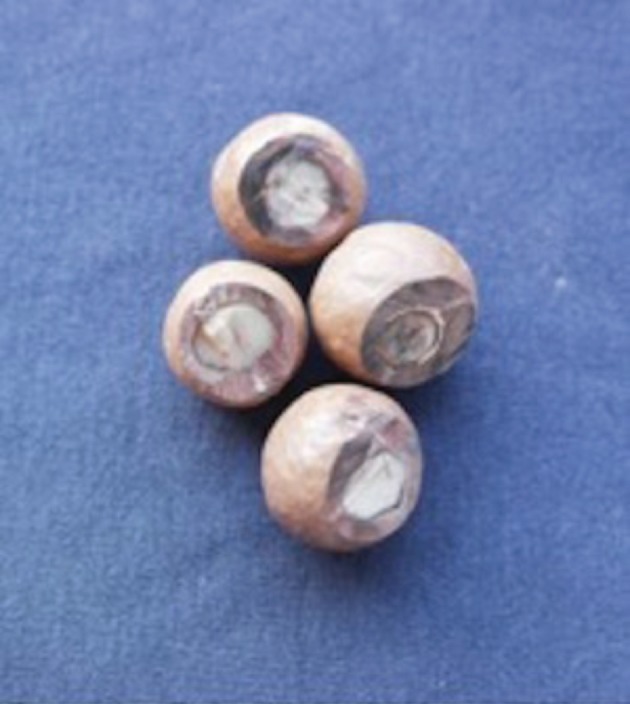
Macadamia nuts with side shaved off as used in the partial end-state condition (Photograph by E.B.).

In the first demonstration condition, the subjects were provided with 15 macadamia nuts that had already been partially opened in the laboratory, outside of the view of the subjects (figure 2). The nuts were still in their shells, but one side was shaved off to allow for the nut inside to be clearly seen, ensuring that the subjects were aware that the edible nut was inside the shell. Thus, this condition, while providing information that the macadamia shells contain edible kernels, did not provide information about hammer usage, or hammer effects (i.e. about the condition of nuts that have been hammered). A further 15 shelled macadamia nuts were provided for the Shepreth Wildlife Park subjects and 15 shelled macadamia nuts and seven shelled sea almonds were provided alongside the shaved demonstration nuts at Curraghs Wildlife Park. Macadamia nuts were always used for the demonstrations. Fifteen macadamia nuts were provided in all conditions for both groups, alongside seven sea almonds in the Curraghs Wildlife Park group. As the subjects were never successful with cracking any nuts, the number of nuts in the enclosure increased with each condition (as 15 new macadamia nuts and seven sea almonds were introduced into the enclosure in each trial).

##### 3.4.2.2. End-state condition

The next demonstration involved cracking nuts outside of the view of the subjects, and placing them back inside their shells. This allowed the subjects to see again that edible nuts were inside the shells, but did not provide information on the hammering action required for the nuts to be opened. Here, subjects were able to remove the nut from the cracked and loose shells to consume the kernel. Fifteen cracked macadamia nuts were placed inside the enclosure alongside the same number of shelled nuts as in the previous condition (15 macadamias and seven sea almonds).

##### 3.4.2.3. Object movement demonstration condition

**Figure 3.**
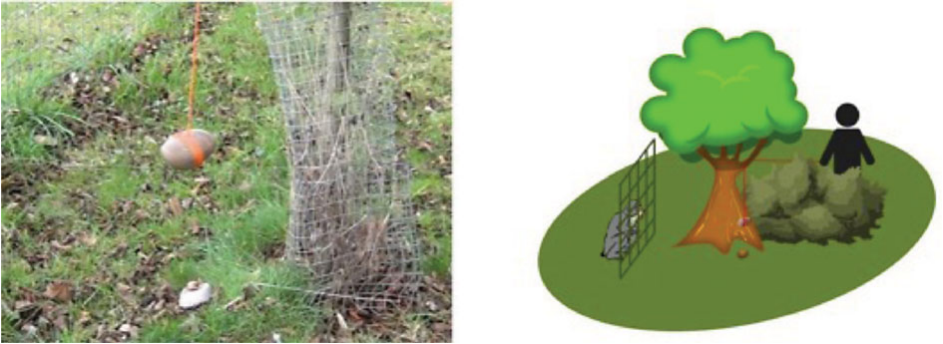
Photograph (by E.B.) and diagram (by D. Neadle) of the pulley system used to demonstrate the cracking action of the stones.

The third demonstration involved an object movement demonstration condition, in which the environmental result of the shell cracking was demonstrated alongside the movements required to crack the nut, but without an active agent carrying out any actions which could be copied (technically this was simultaneously both an object movement demonstration and end-state condition). A pulley system was devised with a stone (size M) attached to a string and draped over a branch of a tree standing in front of the outside area of the enclosure, between the protective barrier separating visitors from the enclosure. The tree was visible to the subjects when in their outdoor enclosure (figure 3). A macadamia nut in its shell was placed on top of the stone anvil and the string to which the stone was tied was released, allowing the stone to fall (from an approximate height of 50 cm) on top of the nut, cracking it open. The open nut was then handed to the subjects through the mesh. Demonstrations were repeatedly carried out for approx. 15 min. Each demonstration lasted between 5 and 10 s (from the release of the stone to the nut cracking). After each demonstration, the cracked nut was handed to the subjects through the mesh and the usual number of shelled macadamia and sea almonds were added (15 macadamias and seven sea almonds).

#### 3.4.3. Action-dependency testing conditions

##### 3.4.3.1. Full demonstration condition

A full demonstration (containing both copy-able pound-hammering actions and the accompanying results (including end results) of stone movement and object cracking) was provided by the keeper (A. Perry) at Shepreth Wildlife Park and by the researcher (E.B.) at Curraghs Wildlife Park. Before each trial, the demonstrator positioned themselves in front of the subjects, and placed a macadamia nut on the anvil and used one of the stones (size S or M) to crack open the nut. The opened nut was then handed to the subjects. Once the individual had consumed the nut, another full demonstration was provided. At Shepreth Wildlife Park, each individual was exposed to three demonstrations before being provided with 15 shelled macadamia nuts. At Curraghs Wildlife Park, demonstrations were provided continuously for approx. 15 min before each trial. Each demonstration lasted between 10 and 25 s. Subjects were then provided with the usual number of shelled macadamia nuts and sea almonds after the demonstrations (15 macadamias and seven sea almonds).

##### 3.4.3.2. Full subspecies conspecific demonstration video condition

To control for the effect of heterospecifics providing the demonstration, videos of wild long-tailed macaques (*Mfa*) provided by field-researchers (L. Luncz, M. Gumert) and taken from online sources, pound-hammering both nuts (sea almonds) and oysters were shown to the subjects before each trial. Videos were played on loop on a Samsung Galaxy Gt-p5110 tablet (833 × 870 mm; 800 × 1280 pixels) for 15 min. The video lasted 28 s in total and contained six cracking episodes per video. Subjects were then provided with the usual number of shelled macadamia nuts and sea almonds (15 macadamias and 7 sea almonds). It was not possible in this study to individually train subjects to show the behaviour and act as a demonstrator for the rest of the group (due to local restrictions at the testing institution and time constraints). However, this condition (in which a live conspecific provides a demonstration of the full behaviour to the rest of the group) would provide a valuable further condition to examine the role of high-fidelity social learning in the development of the target behaviour, and should be carried out in addition to the social learning conditions described above in future, if testing conditions allow.

After all the conditions were carried out, keepers continued to include shelled macadamia nuts in with the subjects' usual daily feed for a subsequent six week period to control for whether a longer period of individual trail-and-error learning might be required for the behaviour to emerge. Keepers reported back to E.B. with the results of observations during this extended testing period.

### 3.5. Coding

All videos were coded following testing. Length of time spent manipulating the nuts was recorded, alongside seven different manipulation types: *carry/hold, gnaw, sniff, hit/drop, roll/rub on hard substrate, roll/rub in hands* (see electronic supplementary material for video of *roll/rub on substrate* and *roll/rub in hands*), *masturbate*. See table 1 for a description of each category.

**Table 1.** Description of manipulation types.

method	description
carry/hold	individual manipulating the nuts either standing or sitting still, or while moving around the enclosures
gnaw	individual uses their teeth to bite and try to crack the nut, or when the nut is inserted into the mouth
sniff	nut is sniffed
hit/drop	nut is either hit with the hand or fist, or dropped from above
roll/rub on hard substrate	nut is rolled or rubbed with the palms on a hard surface, such as the ground, log or stone
roll/rub in hands	nut is rolled or rubbed in between the two hands
masturbate	nut is used to masturbate

Single manipulation types and combinations were coded. Since the subjects at Curraghs Wildlife Park were tested in their groups, there were several occasions of manipulations being interrupted by external factors (the individuals at Shepreth Wildlife Park were tested individually, so did not experience interruptions from other individuals). Therefore, context of manipulation was coded only for Curraghs Wildlife Park. Context was coded as: *antagonistic behaviour*, *groom*, *sex* and *noise*. See table 2 for a description of the contexts.

**Table 2.** Descriptions of the contexts of nut manipulations for Curraghs Wildlife Park.

method	description
antagonistic behaviour	individual manipulating the nut either received an aggressive action or another individual came too close and the manipulating subject performed an aggressive act towards the other macaque
groom	individual manipulating the nut is interrupted by another individual initiating a grooming session
sex	individual manipulating the nut is interrupted by a sexual advance by another individual
noise	individual manipulating the nut is interrupted by an external or internal noise

For the conditions in which demonstrations were provided (object movement demonstration, full demonstration condition and full subspecies conspecific video demonstration condition) all data (including that from Shepreth Wildlife Park) were coded for the eye-gaze (i.e. where the individual was looking, see below) of the subjects during demonstrations, as a measure of attention. However, assessing the eye-gaze of subjects from the videos proved to be difficult due to the fact that the subjects at Curraghs Wildlife Park did not have IDs and could not be individually identified. Furthermore, at both parks, subjects had access to the whole enclosure and moved around continuously during demonstrations, making it difficult to track which individuals had seen the demonstrations before, and how long each individual watched the demonstration. This was especially pronounced at Curraghs Wildlife Park where the individuals were tested as one group. Therefore, only clear cases of directed eye-gaze towards the demonstration (i.e. looking towards the stone falling on the nut in the object-movement demonstration condition; looking towards the researcher or keeper during the full action demonstration or towards the screen during the full subspecies conspecific video demonstration) were coded. An individual was recorded as observing the demonstration when their head (regardless of the direction of the body) was directed towards the demonstration (see electronic supplementary material for a clip of an instance coded as eye-gaze). As mentioned, the individuals at Curraghs Wildlife Park do not have IDs and cannot be individually identified; therefore eye-gaze was coded as instances rather than by individuals. As we could not always be completely confident of the eye-gaze of the individuals, and due to the issues mentioned above, we applied very stringent requirements for the eye-gaze coding. These strict requirements may have resulted in fewer individuals being coded for eye-gaze towards the demonstration than in reality. Similarly, some of the individuals who watched the whole demonstrations may have been excluded from the dataset because they did not fit all the requirements for eye-gaze. However, we opted for the strict instructions for this set of coding in order to avoid false positives.

#### 3.5.1 Reliability coding

Twenty per cent of all the testing videos were second-coded by a blind coder, according to all the behavioural categories outlined in the previous section. There was a strong and substantial agreement between coders for all the behavioural categories (Length of time manipulating nuts: Cohen's kappa; *k* = 0.72; Manipulation types; *k* = 0.81; Context of manipulation; *k* = 0.78). Twenty per cent of the videos were also second-coded for eye gazing by a naive coder and there was a substantial agreement between coders, *k* = 0.71.

## 4. Results

None of the subjects in this study used the stones to crack the nuts in any of the conditions. Thus, the captive long-tailed macaques (*Mff*) neither individually expressed pound-hammering, nor did they socially learn to do so. The subjects were not successful in cracking the nuts using other methods, either. Despite the fact that they were never successful, the interest in the nuts and motivation to open them remained high throughout all testing sessions, demonstrated by a consistent manipulation of nuts across testing sessions. Over 105 h of observational data were collected from both Shepreth Wildlife Park and Curraghs Wildlife Park combined, and in 89% of each testing session at least one subject was manipulating nuts. Manipulation bouts lasted between two seconds and eight minutes. Mean interaction time with the nuts was 0.38 s (s.d. = 1.23).

Seven different manipulations of the nuts were observed: *carry/hold, gnaw, sniff, hit/drop, roll/rub on hard substrate (ground, log or stone), roll/rub in hands* and, on one occasion, using a macadamia nut to *masturbate* (see methods section for full descriptions of each behaviour). Single manipulation of the nuts was recorded in 44.7% of bouts and combination was recorded in 42.1% of bouts. The use of three different manipulation types was recorded in 10.5% of bouts, while using four different types was observed in 2.6% of cases. The most common single manipulation was ‘gnaw’ (57.4%) followed by ‘carry/hold’ (18.5%), ‘roll/rub in hands’ (7.4%), ‘sniff’ (3.7%) ‘roll/rub on hard substrate’ (3.7%), ‘hit/drop’ (3.7%), ‘masturbate’ (1.9%). The most commonly used combination of manipulation types was ‘gnaw & sniff’ (25%), followed by ‘carry/hold & gnaw’ (17.8%), ‘gnaw, roll/rub in hands, roll/rub on substrate’ (14.3%), ‘roll/rub in hands & gnaw’ (14.3%), ‘carry/hold & sniff’ (7.14%), ‘roll/rub in hands & gnaw’ (3.6%), ‘carry/hold, gnaw, roll/rub in hands’ (3.6%), ‘carry/hold, rub/roll in hands, roll/rub on substrate, hit/drop’ (3.6%), ‘rub/roll, carry/hold, hit/drop’ (3.6%), ‘carry/hold & rub/roll in hands’ (3.6%).

At Curraghs Wildlife Park, subjects were tested in a group setting, and the videos were coded for interruptions. Interruptions were frequently due to antagonistic behaviour between individuals. In 48% of manipulations of the nut there was an instance of distraction. Distractions were coded as *antagonistic behaviour, groom*, *sex* and *noise*: the most common interruption was due to *antagonistic behaviour* (79.5%), followed by *sex* (6.9%), *groom* (6.8%) and *noise* (6.8%). The mean length of an interruption was 8 s (s.d. = 2.33). In 34.5% of cases the nut was lost (either stolen by another individual or left behind) as a result of the interruption.

Stones were rarely manipulated throughout the whole experiment. Instances in which a stone was manipulated at the same time as the nut were coded, and in only 9.8% of cases were the stones manipulated at the same time as the nuts. Of these cases, 44% of times the stone was used as a surface to roll the nut on, and in all the remaining cases, the stone was simply held in the free hand or rolled around the enclosure. Only two instances of stone manipulation independently of nut manipulation were recorded, and both involved the stones being moved to investigate the area underneath the stone.

Despite the high levels of motivation (see above) none of the subjects used the stones provided to crack the nuts—or even to attempt to crack the nuts—in either the baseline or any of the social learning conditions. The subjects continued to be unsuccessful during the additional six weeks at the end of testing when the keepers at Curraghs Wildlife Park provided the nuts alongside the subjects' regular feed.

### 4.1. Eye-gaze

Overall, low levels of attention were recorded during all three demonstrations. Subjects only watched on average 2.22% of the whole demonstration session (which lasted 15 min in each demonstration condition). However, when assessing the mean time watching each individual demonstration, subjects watched a higher percentage of the demonstration. The object movement demonstration lasted on average 7.5 s (s.d. = 4.3), of which individuals watched on average 13.3% of each demonstration. Full demonstration lasted on average 18 s (s.d. = 19.4), of which subjects watched 25.9% of each demonstration. Each video in the full subspecies conspecific video demonstration was 28 s long, with subjects watching 9.1% of the videos. Two individuals could be confidently considered to have watched a full demonstration. The first was Tina (F, 25 years), in Shepreth Wildlife Park, who watched one whole full demonstration of the keeper using a stone to crack open a nut. The second instance occurred during the video demonstration in which an individual (M, unknown age) at Curraghs Wildlife Park watched all 28 s of the subspecies conspecific video demonstration (as the video contained six instances of pound-hammering by different individuals, thus this subject can be considered to have watched six demonstrations of the target behaviour). Therefore at least two individuals, one from each park, watched one full demonstration.

## 5. Discussion

Despite ample individual learning opportunities as well as various social learning demonstrations (including the demonstration of underlying actions), pound-hammering did not appear in any of the tested captive *Mff*. The *Mff* macaques in this study did not spontaneously develop pound-hammering individually, but also did not socially learn the behaviour with the help of any of the available social learning mechanisms that our conditions allowed for (i.e. across the partial end-state condition, end-state condition, object movement demonstration condition, full demonstration condition and the full subspecies conspecific demonstration video condition; see Introduction). The sample (*n* = 31) of our study exceeded the power requirements for both the single and the double-case standards [41], allowing us to draw conclusions on a species level from our negative findings (see above). Thus, we conclude that our data do not show that pound-hammering can be individually learnt by *Mff.* This however raises the question of why the behaviour did not emerge in captive *Mff.*

### 5.1. Possible explanations for the lack of pound-hammering in *M. fascicularis fascicularis*

#### 5.1.1. Genetic predispositions

One explanation as to why the behaviour did not emerge in naive *Mff* is that there may be a genetic component that is only found in *Mfa*. This may explain why the behaviour is present in wild *Mfa* but absent (so far) in *Mff*. It may be that *Mfa* have a genetic predisposition for enhanced individual learning and, subsequently, some forms of social learning relevant for the expression of pound-hammering. As the underlying mechanisms for individual and social learning are likely based on associated mechanisms [55–57], one potential explanation for the presence of this behaviour in wild *Mfa* and not in *Mff* (in wild and captive *Mff* populations, such as the one tested here) may be that the two subspecies have differing levels of individual learning abilities and motivation to attend to socially mediated information.

Based on the assumption that individual and social learning had an interdependent evolutionary path [56] (see also [55]), it would seem likely that species that are better at individual learning should therefore also be more attentive to social information. In this study we found that the captive *Mff* demonstrated very low levels of attention to all the social demonstrations provided. Despite the range of social demonstrations, the subjects in this study only watched a maximum of 25% of a demonstration (in the full demonstration condition), and we could only confirm for two individuals that they watched a whole demonstration (note however, as mentioned above, it may be that more individuals watched a whole demonstration but were excluded by the conservative requirements we set for these data). Thus it may be that *Mff* are relatively uninterested in socially mediated information, and, as a result, are also less likely to individually or socially learn the behaviour (overall low levels of attention to social demonstrations were also found in marmosets; a study on the attention of marmosets to knowledgeable demonstrators manipulating a problem-solving task found that individuals only attended to the demonstrator for a median of six seconds [58]). Indeed, a recent study on two different subspecies of otters also found differences between the subspecies in their levels of attention to socially mediated information [59]. However, this study did not directly test the role of genetics in pound-hammering in *Mfa*, and in the absence of data on the levels of attention to social information by wild *Mfa*, it is currently impossible to assess whether a distinct difference in the levels of individual and social learning does indeed exist between the subspecies. Yet, a possible genetic component to the behaviour may provide one explanation as to why wild *Mff* do not show the behaviour, but a population of hybrids of *Mff* and *Mfa* in the wild do show pound-hammering.

Although the *Mff*s showed overall levels of low attention to the demonstrations, it is important to note that at least two individuals did watch at least one full demonstration. One individual, Tina, watched a full human demonstration and one individual from Curraghs Wildlife Park watched the subspecies conspecific video demonstration in full (thus this individual watched six demonstrations of the target pounding behaviour). Therefore, at least two individuals attended to all the social information—including the actions—required to crack open the nuts using stones. Subsequently, if the behaviour required social information to be expressed in the naive macaques, at least the two individuals that attended to the full demonstrations should have been equipped with the knowledge necessary to express pound-hammering. Yet, the behaviour still did not emerge, suggesting either that a longer exposure to social information is required for the behaviour to develop, or, more likely, that social information may not be sufficient to encourage the acquisition of pound-hammering—potentially due to a lack of motivation to use the information, and/or to a lack of imitative ability.

#### 5.1.2. Sensitive learning periods

An alternative explanation for the absence of the target behaviour observed in this study may be that a sensitive period for the acquisition of this behaviour exists early in ontogeny [50,60–62]. Indeed, Tan [50] found that wild juvenile *Mfa* *×* *Mff* hybrids only begin practising pound-hammering and axe hammering at around 3 years of age. The period before the acquisition of this behaviour consists of extensive play and manipulation bouts with the stones and nuts involved in the later behaviour [50]. Tan [50] concludes that this extended period of manipulation of the objects is required for the full behaviour to emerge in adulthood. A similar finding was reported for juvenile chimpanzees, who may only acquire nut cracking after a sensitive period in which they manipulate the materials of the behaviour between the ages of 3–5 years, and an extensive trial-and-error learning period between 8–14 years in which they perfect the technique [61]. Although the subjects in this study ranged from infants to older adults and all ages were represented, it is possible that a long period of manipulation of the stones and the nuts while in the sensitive learning period is required for the behaviour to emerge. Here we provided all the materials in the subjects' enclosures and daily feed for a total period of four months, with no reports of the behaviour emerging even after this extended exposure to the nuts. However it might be that up to 3 years of exposure to the materials is required before the behaviour develops. Thus, the absence of the materials within this extended sensitive learning period might have limited the development of pound-hammering observed in this study.

#### 5.1.3. Motivation levels

It might also be that the individuals were not motivated enough to solve the task, but this seems unlikely for our subject sample as the levels of manipulation of the nuts remained high throughout the whole testing period (and keepers reported that the macaques continued to try to open the nuts even after testing). However, it is possible that the two Shepreth Wildlife Park *Mff* individuals' rejection of clams (provided in the first test) reflects a general dislike of molluscs in this subspecies, which may be one of the factors limiting the emergence of pound-hammering in both wild *Mff* and our captive population. Pound-hammering is observed primarily in coastal areas in which *Mfa* have access to marine shelled foods, which they consume more than other encased food sources, such as nuts [6]. Indeed, observations of wild *Mfa* cracking nuts have only recently increased, perhaps also as a response to the increase of palm oil monocultures in their environment [5,54]. Thus, it could be that pound-hammering emerged primarily to exploit marine encased food sources and was only after then generalized to cracking nuts (M. Gumert 2018, personal communication). Thus, if *Mff* are not interested in cracking open molluscs and are not motivated enough to open encased nuts as they have access to other food sources, they may have not developed the tool-use abilities to exploit any encased food sources***.*** This explanation seems more likely than one that suggests that *Mff* cannot use tools at all, as both captive and wild long-tailed macaques have already been found to spontaneously show tool-use behaviours. For example, Zuberbuhler *et al*. [44] describe the spontaneous emergence of a raking behaviour to retrieve out of reach apples from outside the enclosure in one *Mff* individual (suggesting this to be an individually learnt behaviour, although note that this single observation does not fulfil the double-standard required of relatively less complex behaviours, of which we believe this to be a case), and there have been other observations of sporadic tool use in wild *Mff* [63,64], including one observation of stone tool use [65]. Thus, it seems that long-tailed macaques are likely to at least possess the motivation and capability to spontaneously learn some tool-use behaviours, making the absence of pound-hammering in our study all the more surprising. However, it may be that the cognitive requirements for multi-step stone tool-use behaviours, such as pound-hammering, are different from those required for more general tool use, and that although *Mff* can spontaneously express simple tool-use behaviours, more complex stone tool behaviours are at the limits of their learning abilities.

#### 5.1.4. Pre-existing techniques

Another possible explanation for the lack of emergence of this behaviour in the naive macaques is that once a strategy to retrieve a specific resource is acquired, it might negatively impact the emergence of related strategies in that individual. For example, if an individual has already learnt to use a specific tool or technique to retrieve honey from a tree, this pre-existing strategy may hinder the individual's ability or motivation to innovate a different method to retrieve the same food source (e.g. [66,67]; comment on [68]). A relative inflexibility in switching methods (and/or lack of motivation to do so) may have also played a role in the current study. Before testing, the macaques only received shelled nut types which they could crack open with their teeth or by dropping them from elevated surfaces (e.g. coconuts; interestingly, this dropping technique was only observed in 3.7% of manipulations with the macadamia nuts and sea almonds, perhaps due to the fact that it was never successful with the nuts we used here). The most commonly observed manipulation type recorded across both groups of macaques in our study was the ‘gnaw’ manipulation (57.4%), which involved the individuals trying to crack the nuts open with their teeth. As this gnawing strategy worked in the past with other types of nuts, it may be that the macaques were not able to switch to a new technique, even if gnawing became inefficient (impossible as a solution) in our study.

We also observed the macaques in this study to adopt a ‘rolling’ manipulation, in which the individuals would roll or rub the nut between their hands or on a hard substrate, such as a rock or piece of wood (this occurred in 7.4% and 3.7% of manipulation events respectively). Rolling or rubbing the nuts never resulted in the opening of a nut. However, this rolling behaviour has also been observed in wild Balinese *Mff*, who rub objects such as seeds, empty shells (coconut and snail shells), peanuts, sweet potatoes, rocks and insects such as caterpillars and worms between their hands before eating or abandoning the object [64]. The rolling of food sources in the wild *Mff* has no apparent purpose, as it does not seem to help with the opening of the food source (if it is encased) and is often carried out also with inedible objects (such as rocks and shells) or already dead animals (such as caterpillars and worms) [64]. Therefore, in addition to the gnawing behaviour, it might be that these two behaviours negatively impact the exploration of hammering strategies.

### 5.2. The role of social learning in the emergence of tool use

Given the widespread occurrence of diverse social learning mechanisms across animal species [69], it is reasonable to assume that primate behaviours observed in the wild are also influenced by social learning (at least in increasing the *frequency* of some behaviours; see also [41]). However, in the captive population of *Mff* tested here social information was not sufficient to elicit the emergence of the target pound-hammering behaviour. This is not the first study to find that social learning did not encourage the emergence of a behaviour in individuals who did not spontaneously express it in the first place. In their study on tool use in naive woodpecker finches, Tebbich *et al*. [60] found that all of the juvenile naive finches in their sample expressed a wild tool-use behaviour (the behaviour involved using twigs to retrieve beetle larvae from an artificial tree trunk) without social learning. On the other hand, some of the adults in their group did not develop the target behaviour and exposing these adult finches to tool-using models also did not increase their likelihood of the behaviour emerging either (thus perhaps suggesting that a sensitive learning period may exist for the acquisition of this behaviour, see above). Similarly, Visalberghi [9] observed two capuchins (*Cebus apella*) spontaneously cracking nuts. The behaviour was not exhibited by the rest of the group, despite the tests being carried out in a group setting (with all ages represented), allowing for ample opportunities for the rest of the group to observe the two nut-cracking capuchins and thus for social learning to take place. Kenward *et al*. [70] ran a study in which they found that two juvenile hand-raised New Caledonian crows that had never been exposed to tools or demonstrations on tool-making, spontaneously made twig tools to retrieve food from a crevice. Two other crows, also hand-raised, were provided with full action demonstrations on how to make the tools, but the authors found no difference in tool-oriented behaviours between the naive crows and the ones that had received demonstrations [70]. Thus, it seems that, similar to what was found in the current study, social learning may not always be the key to release (or even copy) the behaviour; even in behaviours that can and are individually learnt (although note that most bird species have social systems different from chimpanzees, which should be taken into account when comparing the individual and social learning abilities of birds and primates).

It might be argued that human demonstrators are not efficient models for non-human animals [71], and that perhaps the reason why the behaviour was not socially facilitated in the human full demonstration condition might have been because the subjects in this study did not recognize the human demonstrators as efficient social learning models. However, evidence for the view that only conspecifics are valuable demonstrators is limited (e.g. [27], in which the actions of a novel behaviour were not copied even when they were demonstrated by a conspecific, and see also the results of ghost demonstrations in which chimpanzees expressed the target behaviour even without any demonstrator; e.g. [32]). Yet, to control for this potential confound, (video) demonstrations from subspecies conspecifics were provided in our study. Although video demonstrations are not as effective as live conspecific demonstrations [32], previous studies have found that video demonstrations can influence the behaviour of observers (e.g. [72,73]). Due to local restrictions at the testing institutions, the fact that *Mfa* are not currently found in captivity and that the *Mff* in our sample never showed the behaviour, or even precursors of the behaviour, it was impractical to train a live conspecific demonstrator in this behaviour (and even if *Mfa* individuals did exist in captivity, we would have had to introduce a demonstrator of a different subspecies into the *Mff* group), thus videos of unfamiliar *Mfa* conspecifics showing the pound-hammering behaviour were provided. The videos were, however, the least-watched demonstrations (9.1% of each video was watched) and the behaviour did not emerge after this condition either, suggesting that having subspecies conspecifics demonstrate the behaviour did not have an effect on the likelihood of expression of the behaviour. Future studies should focus on attempting to train *Mff* individuals to provide live demonstrations of the actions required for pound-hammering to the rest of their group, to observe whether this type of demonstration helps release the behaviour (however, given the results of the social learning demonstrations provided in this study, we believe this to be unlikely). The outstanding question on why pound-hammering has emerged in wild *Mfa* communities, and not in *Mff* populations therefore remains. The *Mff* subjects in this study did not spontaneously use tools to crack open the nuts, nor did they learn the behaviour from various demonstrations (including demonstrations from other long-tailed macaques, and demonstrations of the underlying behavioural form).

While the reasons behind the lack of tool use in our population of captive *Mff* remain inconclusive, this paper provides a new methodological approach, including a method to calculate the minimum sample sizes required, to examine the learning mechanisms behind the development of tool-use behaviours that can be applied across animal species. By providing both an asocial baseline and several levels of social learning conditions, the roles of each learning mechanism can be identified in the emergence of novel behaviours. This methodology can also be used to experimentally test the ZLS hypothesis for particular behaviours, as it argues that many animal tool-use behavioural forms are the product of individual, rather than social, learning [23,41]. The ZLS approach favours an individual learning based approach, in which behavioural forms within a species ZLS can emerge via individual learning, with low-fidelity social learning playing a facilitating role in increasing the frequencies of these behaviours across populations (e.g. [37–42,74]). The results of this study do not, however, support the ZLS hypothesis for the tested behavioural form as the naive *Mff* did not individually learn this behaviour. Yet the macaques *also* did not socially learn this behaviour. Thus, future work remains to test more groups of captive *Mff*, following the new methodological approach described in this paper, to examine whether pound-hammering will emerge in any of the individual or social learning conditions in other populations. Furthermore, the roles of genetic predispositions, sensitive learning periods, levels of motivation and pre-existing techniques in the emergence of pound-hammering should be further investigated in both subspecies of long-tailed macaques.

## Supplementary Material

Supplementary Information

## Footnotes

1 In these cases of low-fidelity social learning, the individual drives the production of the form of the behaviour, with low-fidelity social learning merely providing a stimulus/extra motivation for this process. Thus, these cases of social learning may also be described as ‘individually derived behaviours (in cultural disguise)’.

2 The obvious downside to this method is that it may induce carry-over effects between conditions. However, here we deemed this a smaller disadvantage than the alternative option of having diminished sample sizes that would have resulted from a between-subjects design. Note that *any* order in which we applied these LS test conditions still allowed us to test for the necessity of observing the target behavioural form demonstrations for the expression of the behaviour in subjects, regardless of possible carry-over effects.

3 These would be behaviours that are hardwired, i.e. they would be close to the now out-dated concept of ‘instinct’.

## Data accessibility

All relevant data are included in the electronic supplementary material section. This includes a full explanation of the cumulative binomial distribution formula used to calculate the required sample sizes for the single and double-case ZLS standards and our calculations in a stepwise approach. The wildlife parks where this study was conducted have asked us to not publically share the data collected for this study. However, they have agreed to clips of the relevant behaviours to be shared. Therefore, we have uploaded as part of the electronic supplementary material video clips of the relevant behaviours observed in the subjects of this study, including a clip of an event coded as ‘eye-gaze’.
