# Supplementary material for: Naive, captive long-tailed macaques (Macaca fascicularis fascicularis) fail to individually and socially learn pound-hammering, a tool-use behaviour
Source: R Soc Open Sci. 2018 May 9;5(5):171826. doi: 10.1098/rsos.171826 (PMC5990768; doi:10.1098/rsos.171826)
Supplement: Supplementary Information [file rsos171826supp1.docx]

Supplementary Information

*Binomial Cumulative Distribution*


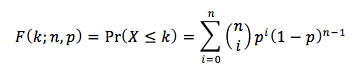

in which: $\left( \frac{n}{i} \right)$= n!/i!(n-i)!

To figure out the probability of two or more inventions, first the likelihood of 0 inventions, and then 1 invention has to be calculated. Adding the two, and subtracting the sum from 1, the likelihood of 2 or more inventions can be found.

F(k;n;p)=what follows is a function where k;n;p =Pr(X<-k)= probability of invention (X) is minus, or equal to k, and:

$\left( \frac{n}{i} \right)$= n!/i!(n-i)!

For the case of 31 macaques (sample size) and 0.10 reinvention probability, first the likelihood of zero reinventions is calculated:

$\sum_{i=0}^{n} \left( \frac{n}{1} \right)$in which (n)=is the floor

**Pr (X=k)=(31!/(0!(31-0!))) (.10^0^(1-.10)^31-1^) + (31!/(1!(31-1!))) (.10^1^(1-.10)^31-1^)**

Zero equation first:

n!/i!(n-i)! = 31!/0!(31-0)! , where 0! Is always = 1

31!/1(31-0)! = **1**

So,

(1).10^0^ (1-.10)^31-1^ = (1) 1 (1-.10)^30^ **= 0.0424**

So,

0.0424 + (31!/(1!(31-1!))) (.10^1^(1-.10)^31-1^)

n!/i!(n-i)!=31!/1!(31-1)! = 31!/1!(30)! = 31!/(30)! = 31

31 (.10^1^) (1-.10)^30^ = 31 (.10) (1-.10)^30^ = **0.1314**

So, 0.0424 + 0.1314 = **0.1738**

And the likelihood of two or more inventions is: 1-0.1738 = **0.8262**

**Table of minimum sample sizes for each of the ZLS standards**

|  | Double-Case ZLS | Single-Case ZLS |
| --- | --- | --- |
|  |  |  |
| 0.10 innovation probability (80% power) | **Min. *n=* 29**  (Power for *n=*28: 78.4) | **Min. *n=* 16**  (Power for *n=*15: 79.4) |
